# Dental Operations

**Published:** 1856-10

**Authors:** 


### DENTAL OPERATIONS.

In looking over our dental journals we find *almost* all subjects, connected either directly or indirectly with dental practice, written upon. Any thing strange or new, is exhibited full length.

Some most difficult and rare operation that would not occur to one operator in fifty, once in half a professional life-time, will be most minutely and elaborately dilated upon, quoted, commented upon and talked of by every one, just as though such cases constituted the principal part of dental practice.

Emanations from the fertile brains of inventive geniuses are treated in the same manner, and too often without respect to their value. Now, we are not complaining about all this, but we have something to say of one or two things, nevertheless.

While the wonderful, the difficult, the rare and new receive their due proportion of consideration, the common, every-day operations, that *make* up the dental practice, are almost wholly overlooked, save in our valuable text works.

Who ever thinks of giving, for the benefit of the profession, his method of filling a crown cavity of a molar tooth, describing clearly and distinctly each step in the operation; and then extending his description to the various forms and conditions which even these decays may assume, and how all the attendant difficulties are met? Now it is by a close and thorough consideration and investigation of these common, every-day operations, that the profession is elevated. It is this course, coupled with untiring perseverance, that has made our best operators. It is by concentrating the mind upon this kind of operations that any one will excel. After that which is most frequently required is perfectly accomplished, then the more rare and difficult may be pursued. How common do we hear the remark, "I am anxious to witness a certain operation; it is said to be a very difficult one. I have never performed it, or at least to my satisfaction. Now any ordinary operation I can please myself exactly, and I think if I can just see that performed *once* I can—can." Yes if you can just *see* it performed *once*,—"powerful ingenious man"—you can perform it as satisfactorily as you did before, or *perhaps* as satisfactorily to yourself as you do your *common* operations. Any one who is "exactly pleased" with his common operations, *is a superior* operator, or has an unbounded amount of egotism, with a considerable admixture of *wisdom* and *knowledge* of a negative character. Our *best* operators have availed themselves of all the advantages extant for the *perfect* performance of the common operations.

I have observed that our superior operators are constantly inquiring, "How *can* we make *more perfect* our every-day operations?"

We might refer to an instance or two. Dr. J. S. Clark, of New Orleans, by perseveringly keeping his mind in this channel, availing himself of the experience of others, and the various professional advantages with which he was surrounded, has given to the profession an advance step, in the operation of filling, that has scarcely a parallel. I refer to the method of filling with cylinders or blocks—a method by which from one fourth to one third more gold can be introduced into a cavity than by any former mode, and in every respect makes far better fillings. Dr. Clark will please pardon me for this reference to him. I wanted an illustration, and I could not find a better.

Now there is not a man in the profession who, if he would pursue such a course (we refer to those who have not hitherto done so), would not be astonished at the results.

Now, gentlemen, professional brothers! In the operation of filling, do you make every operation, and each step of every operation, as perfect as *you can?* Are your excavators and pluggers all of the most approved patterns—perfectly adapted to the purpose for which they are designed, of the most perfect material, and in the best possible condition in regard to temper, sharpness of edge, etc.? Have you the best material for filling and prepared in the best manner? Have you good new files, and sharp cutting instruments? or are your files filled with bone and covered with the blood of fifty patients? Do you bring the teeth and contiguous parts to the best possible condition; or do you go to work as you would upon an inorganic body, ignoring everything like physiology, pathology, nerves, sensibility, or the possibility of a diseased condition ever arising?

In approximal decays, do you make ample and proper separations? and in crown cavities, do you open up the cavity as it should be, removing the projecting portions of enamel? Do you always give the cavity the best possible form, with the least loss of dentine? How often is from one to two hours consumed in skillful manipulations to form a cavity, examining and forming perfectly *every point* in the cavity? Do you aim to introduce your gold so that the greatest possible amount will be put in, and also to restore as nearly as possible the original form of the tooth? Do you perform every step in the common operations, so that it could not be improved? Do you employ the time and labor to do it?

It is only a rigid regard to all these points, accompanied with a thorough knowledge of the collateral sciences, physiology, pathology, chemistry, therapeutics, etc., and a knowledge of the methods of others, together with practice, that suffice to make a competent operator.

If the best operators in the profession, after having availed themselves of all facilities in the way of appliances and instruments, the experiences of others communicated through the periodicals, and by frequent intercourse, private and public, are only able to make good operations at the expense of great labor, perseverance, and time, how can any one, with but few or none of these advantages, presume to intimate that *his operations* are perfect or even *good.* T.

